# Combined Transplantation of Adipose Tissue-Derived Stem Cells and Endothelial Progenitor Cells Improve Diabetic Erectile Dysfunction in a Rat Model

**DOI:** 10.1155/2020/2154053

**Published:** 2020-07-03

**Authors:** Qiyun Yang, Wanmei Chen, Chi Zhang, Yun Xie, Yong Gao, Cuncan Deng, Xiangzhou Sun, Guihua Liu, Chunhua Deng

**Affiliations:** ^1^Department of Urology, The First Affiliated Hospital of Sun Yat-sen University, Guangzhou 510080, China; ^2^Department of Anesthesiology, The First Affiliated Hospital of Sun Yat-sen University, Guangzhou 510080, China; ^3^Reproductive Medicine Center, The Key Laboratory for Reproductive Medicine of Guangdong Province, The First Affiliated Hospital of Sun Yat-sen University, Guangzhou 510080, China; ^4^Reproductive Medicine Research Center, The Sixth Affiliated Hospital of Sun Yat-sen University, Guangzhou 510655, China

## Abstract

Erectile dysfunction (ED) is a common complication in men suffered with diabetic mellitus. Stem cell transplantation is a promising strategy for the treatment of diabetic ED (DED). In this study, we evaluated whether combined transplantation of adipose tissue-derived stem cells (ADSCs) and endothelial progenitor cells (EPCs) could improve the erectile function of the DED rat model. DED rats were induced via intraperitoneal injection of streptozotocin (50 mg/kg), and ED was screened by apomorphine (100 mg/kg). DED rats were divided into 4 groups (*n* = 14 each): DED, ADSC, EPC, and ADSC/EPC group. Another 14 age-matched male SD rats with normal erectile function were served as the normal group. The normal group and the DED group were received intracavernous injection with phosphate-buffered saline (PBS). And the other groups were received intracavernous injection with ADSCs (1 × 10^6^), EPCs (1 × 10^6^), and ADSCs/EPCs (0.5 × 10^6^/0.5 × 10^6^), respectively. The total intracavernous pressure (ICP) and mean arterial pressure (MAP) were recorded at day 28 after injection. The endothelium, smooth muscle, and penile dorsal nerves were assessed within cavernoursal tissue. On day 28 after injection, the ADSC/EPC group displayed more significantly enhanced ICP and ICP/MAP than the DED or ADSC or EPC group (*p* < 0.05). Immunofluorescent analysis and western blot demonstrated that the improvement of erectile function in the ADSC/EPC5 group was associated with increased expression of endothelial marker (CD31) and the correction of eNOS-cGMP-NO signaling. More 5-ethynyl-2′-deoxyuridine- (EdU-) positive EPCs could be found lining in the cavernous endothelial layer in the ADSC/EPC group than the EPC group, which was attributed to the paracrine of vascular endothelial growth factor (VEGF) and stromal-derived factor-1 (SDF-1) by ADSCs. Combined transplantation of ADSCs and EPCs has a synergic effect in repairing the endothelial function of DED rats, and the underlying mechanism might be the paracrine of VEGF and SDF-1 by ADSCs, which improves the recruitment and proliferation of EPCs in the cavernosum.

## 1. Introduction

Erectile dysfunction (ED), which is defined as an inability to obtain and/or sustain sufficient penile erection to achieve satisfactory sexual intercourse, is a common and depressing complication in men suffered with diabetes mellitus [1]. It is reported that about 35%~90% of diabetic men suffering from ED, which is 3 times higher than the healthy men [2]. Although phosphodiesterase type 5 inhibitors (PDE-5Is) are the first-line treatment for ED nowadays, the response rate in the diabetic ED patients is low [3], mainly because of the severe damaged of cavernousum endothelial function, then subsequently decreasing of smooth muscle content and neuropathy [4]. It is therefore urgent to explore novel strategies for the regeneration of cavernousum, both morphologically and functionally.

Stem cells (SCs) are now considered one of the promising strategies for diabetic mellitus erectile dysfunction (DED) [5]. So far, various stem cells, e.g., mesenchymal stem cells (MSCs) [6], adipose tissue-derived stem cells (ADSCs) [7, 8], and urine-derived stem cells (USCs) [9] have been proven to be effective in the treatment of DED. Among them, ADSCs, which can be obtained by the minimal invasive method and be easily expanded, are thought to be an ideal candidate for the treatment of DED. And the paracrine effect is considered to be the major mechanism for ADSCs in the therapeutic effect of DMED. In our previous study, ADSCs genetically modified with VEGF-165 displayed a greater therapeutic effect in improving erectile function of DED rats than unmodified ADSCs [8]. However, taking into account the risk of the exogenous gene integrating into host genome, transgenic technology is still restricted in clinical application.

Endothelial progenitor cells (EPCs), especially the late-out growth EPCs, which can give rise to mature endothelial cells (ECs) *in vitro* and *in vivo*, are critically important in the regeneration of the injured vessel *in vivo* [10, 11]. Several preclinical studies have demonstrated the significant improvement of endothelial function by EPC transplantation in the hind limb ischemic [12] and coronary ischemia animal model [13]. Studies have suggested that the number of EPCs in DM patients is lower compared to healthy men, and EPC functions were also considerably impaired by DM [14]. The proliferation and differentiation of late-out growth EPCs need various proangiogenic factors, such as VEGF, angiongenin, and angionpioetin I, but these EPCs can barely secrete these factors themselves [15]. Moreover, research also indicated that the expression of VEGF and its receptor decreased within the cavernous tissue of DED rats [8]. Gao et al. found that transplanting EPCs genetically modified with VEGF-165 can partially restore the erection function of DED rats [16].

Recent studies indicated that combined transplantation of MSCs and EPCs could enhance the bone generation and cardiac repaired after myocardial injury [17, 18]. Therefore, we first hypothesize that the paracrine effect of ADSCs can enhance proliferation and differentiation of EPCs; thus, combined transplantation of these cells can display a synergistic effect on improving erectile function and restoring the cavernous structure in DED. In the present study, we will investigate the efficacy of combined transplantation of ADSCs and EPCs in the treatment of a diabetic ED rodent model and the underlying mechanisms.

## 2. Materials and Methods

### 2.1. Ethics Statement

Four 2-week-old male Sprague-Dawley (SD) rats (60 g-80 g) and seventy-five 8-week-old male SD rats (280 g-320 g) were purchased from the Animal Center of Sun Yat-sen University (Guangzhou, China) and kept under a standard laboratory condition. The whole procedures were approved by the Sun Yat-sen University Health Sciences Institutional Review Board.

### 2.2. Isolation and Cultivation of EPCs

Rat EPCs were isolated and cultured according to the previously described, with minor modifications [19]. Briefly, after collecting the mononuclear cells from the tibia and femoral bone marrow of 2-week-old rats, cells were diluted with phosphate-buffered saline (PBS), placed onto Ficoll-Paque Plus (GE Healthcare Bio-Sciences, Pennsylvania, USA), and centrifuged at 18°C at 400 g for 30 min. Then, collecting the Buffy coat mononuclear cells and washed twice with PBS. After that, the cells were resuspended with the EGM-2 Bullet Kit system (Lonza, Basel, Switzerland), which consists of endothelial basal medium, 10% fetal bovine serum, hEGF, hFGF-B, VEGF, IGF-1, heparin, and ascorbic acid. Cells were plated on fibronectin (Millipore, Billerica, MA, USA) coated onto tissue culture plastic. 24 hours after the initial plating, the medium was exchanged to remove the nonadherent cells and was exchanged every day for the first week. Colonies of EPCs appeared 7–10 days after the initial isolation, when the cells grew to 80% confluence and were serially passaged onto fibronectin–coated surfaces. EPCs were used at passages 4 for all in vitro and in vivo experiments.

### 2.3. Isolation and Cultivation of ADSCs

The isolation and culture procedures were followed according to our previously described [20]. Briefly, after collecting from the 2-week-old SD rats, the adipose tissue was incubated in 0.1% collagenase Type I A (Sigma-Aldrich, St. Louis, MO, USA) for 45 minutes at 37°C and shaken 30 seconds at every 15 minutes. After filtration with 100 *μ*m cell strainer and centrifugation, the adipose tissue stromal-vascular fraction (SVF) was immersed in ACK Lysis Buffer for 10 minutes at room temperature. After centrifugation and rinsing with PBS twice, the remaining cells were resuspended in Dulbecco's modified Eagle's medium/F12 media (DMEM/F12, Hyclone, Thermo Scientific, CA, USA) with 10% fetal bovine serum (FBS, Gibco, Life Technologies, USA) and cultured in 25 cm^2^ cell culture flask. The medium was exchanged every other day, and cells were passaged at approximately 80% confluence.

### 2.4. In Vitro Studies

#### 2.4.1. Flow Cytometry Analysis of ADSCs and EPCs

Cell surface antigens of ADSCs (passage 3) or EPCs (passage 3) were determined by flow cytometry analysis. After trypsinized and washed, 5 × 10^5^ ADSCs or EPCs were resuspended in flow cytometry buffer. ADSCs were incubated with fluorescence-conjugated antibodies (CD73, CD90, and CD105), and EPCs were incubated with antibodies (CD 31, CD34, CD45, CD73, CD90, CD105, CD117, and CD133) (BD Biosciences, Franklin Lakes, NJ, USA) on ice for 30 min, respectively. Cell aliquots were then rinsed twice with PBS and detected by flow cytometry (FACS Calibur, BD Biosciences, NJ, USA), and the data were analyzed with FlowJo vX software (Tree Star, Ashland, OR).

At passage3, EPCs were fixed for the endothelium-specific markers (CD31, vWF, and KDR) (Sigma-Aldrich, St. Louis, MO, USA) immunofluorescence staining. And human umbilical vein endothelial cells (HUVECs, passage 3) were served as positive control.

#### 2.4.2. VEGF and SDF-1 Level in the Conditioned Medium of ADSCs In Vitro

VEGF and SDF-1 levels in the conditioned medium of ADSCs in vitro were measured using the VEGF ELLISA kit (RRV00, R&D Systems, Minnesota, US) and the SDF-1 ELLISA kit (R&D Systems) according to the manufacturer's protocol.

#### 2.4.3. In Vitro Tube Formation of EPCs, ADSCs, and ADSCs/EPCs

Matrigel (50 *μ*L per well; BD Biosciences, USA) was first added to 96-well plates and then incubated at 37°C for 1 h. 5 × 10^3^ EPCs, 1 × 10^4^ EPCs, 1 × 10^4^ ADSCs, or 5 × 10^3^/5 × 10^3^ ADSCs/EPCs were plated separately in 50 *μ*L of DMEM and cultured at 37°C with 5% CO_2_ for 2 h; then, the tubes formed of Matrigel were counted under a phase contrast microscopy (Nikon, Japan). The EPC/VEGF group contained 5 × 10^3^ EPCs and 50 ng/mlVEGF in DMEM.

#### 2.4.4. Coculture Assay

Coculture assay was taken in a transwell chamber apparatus with a polycarbonate membrane, according to the manufacturer's instructions. Briefly, the EPC suspension (2 × 10^6^ cells/ml) in EBM-2 was added to the upper compartment of the chamber, whereas ADSCs in DMEM/F-12 containing 10% FBS or the medium alone was loaded in the lower compartment of the chamber. The coculture system was maintained in a humidified atmosphere containing 5% CO_2_ and 95% air at 37°C. The medium was exchanged every other day. After 14-day incubation, the effect of ADSCs on EPC viability was detected by 3-(4, 5-dimethylthiazol-2-yl)-2,5-diphenyltetrazoliumbromide (MTT) assay in the coculture system on days 1, 7, and 14. EPCs were supplemented with 10 *μ*l MTT (Beyotime Institute of Biotechnology, Haimen, China) and incubated for 4 h. After discarding the supernatant by aspiration, 200 *μ*l dimethyl sulfoxide (DMSO) was added to solubilize the purple formazan for 30 min. Then, the OD value was measured with a spectrometer at 490 nm.

### 2.5. *In Vivo* Studies

#### 2.5.1. Establishment of a Diabetic Erectile Dysfunction Rat Model and *In Vivo* Transplantation

The diabetic erectile dysfunction (DED) rat model was induced by streptozotocin (STZ) according to the methods established in our previous studies. Briefly, all rats received one dose of STZ (50 mg/kg) (Sigma, St. Louis, Missouri) via intraperitoneal injection after acclimatization for one week. 2 days after STZ injection, blood samples were obtained from tail prick for random blood glucose measurement with a blood glucose meter (Roche, Mannheim, Germany). Those rats which blood glucose level higher than 300 mg/dl were selected as diabetic rats. Twelve weeks after STZ injection, apomorphine (APO, 100 *μ*g/kg) (Sigma) was used to identify the ED rats according to Heaton's method [21]. After the APO subcutaneous injection, 56/65 (86.15%) rats were screened as DED rats.

After the rats were anesthetized with pentobarbital sodium (40 mg/kg) via IP, a total of 1 × 10^6^ ADSCs or EPCs or 5 × 10^5^/5 × 10^5^ ADSCs/EPCs suspended in 200 *μ*l PBS or 200 *μ*l PBS alone was injected into the corpus cavernosum at the middle level. EPCs were incubated with EdU (Invitrogen, Carlsbad, CA, USA) for 48 h before transplantation. An elastic band was fixed at the base of the penis before the injection of cells and was maintained for 2 min after the cell injection.

DED rats were randomly divided into 4 groups (n = 14 each): ADSC group rats received an intracavernous injection of ADSCs (1 × 10^6^ cells); the EPC group received an injection of EPCs (1 × 10^6^ cells); the ADSC/EPC group received an injection of ADSCs/EPCs (5 × 10^5^/5 × 10^5^ cells); and the DED group received an injection of 200 *μ*l PBS as a negative control. Furthermore, a normal group consisted of 14 age-matched normal rats were served as a positive control.

#### 2.5.2. Erectile Function Evaluation

Erectile function was determined by total intracavernosal pressure (ICP) and mean arterial pressure (MAP) at the 4th week after intracavernous injection as previously described. Briefly, rats were first anesthetized with pentobarbital sodium (40 mg/kg, IP). Then, a PE-50 tube filled with heparinized saline (250 IU/ml) was then cannulated into the isolated left carotid artery to monitor the MAP. For the ICP recording, a 25G needle was inserted into the left penile crus and then connected to another pressure transducer. When the cavernous nerve (CN) was identified and isolated, a bipolar hook electrode connected to a signal generator (Taimeng, Chengdu, P.R. China) was placed to the left CN for electrical stimulation with monophasic rectangular pulses. The stimulus parameter settings were 5 v, 20 Hz, 0.2 ms width, and duration of 60 s. The pressure was recorded and analyzed with the BL NewCentury 2.1 software (Taimeng). To normalize for variations in systemic blood pressure, the erectile function was presented as the ratio of ICP/MAP. Total ICP was measured by the area under the ICP curve according to the method described in the literature. Then, rats were sacrificed with over dose pentobarbital sodium via IP, and the penis was harvested for histological analysis and western blotting.

#### 2.5.3. Histological and Immunohistological Analysis

For Masson's trichrome staining, the prepared paraffin sections were prepared according to our previous published study. Then sections were processed according to the standard protocol of Masson's trichrome staining. Quantitative image analysis was analyzed by the Image-Pro Plus 6.0 software (Media Cybernetics, Bethesda, MD, USA).

For immunofluorescence, frozen penile tissue sections were incubated with primary antibodies to CD31 (Abcam, Cambridge, UK), nNOS (Abcam), or *β*-Tubulin (Cell Signaling Technology, MA, USA) overnight at 4°C, while the control sections were incubated without the primary antibodies. After washing with PBS three times, the sections were incubated with the Alexa-594 or Alexa-488 (goat anti-mouse or goat anti-rabbit, Invitrogen, Carlsbad, CA, USA) at a 1 : 200 dilution for 1 hour at room temperature protecting from light. Then, the sections which had been incubated with CD31 were incubated with freshly configured Click-iT reaction cocktail containing Alexa-488 for 30 min at room temperature without light. Then 4′,6-diamidino-2-phenylindole (DAPI; Invitrogen, Carlsbad, CA, USA) staining was applied for the visualization of cell nuclei. The sections were recorded and photographed with a Leica microscope and analyzed by the Image-Pro Plus 6.0 software.

#### 2.5.4. Western Blotting Analysis

Proteins were extracted from the penis, and concentrations were analyzed by the BCA Protein Assay kit (Beyotime Institute of Biotechnology). Then, equal amounts of proteins were loaded on a sodium dodecyl sulfate polyacrylamide gel for electrophoresis. After transferring the proteins to nitrocellulose membranes, primary antibodies were incubated overnight at 4°C. GAPDH (Proteintech, Rosemont, IL, USA) was used as loading control. Signals were obtained with the Odyssey infrared imaging system. The primary antibodies included CD31 (Abcam, Cambridge, UK), eNOS (Abcam), VEGF (Cell Signaling Technology), and SDF-1 (Santa Cruz Biotechnology, CA, USA).

#### 2.5.5. NO Level and cGMP (Cyclic Guanosine Monophosphate) Level Assay

NO level and cGMP level in the cavernous tissue in each group were measured using a Nitric Oxide Colorimetric Assay Kit (K262, Biovision, San Francisco, US) and a cGMP direct immunoassay kit (K372, BioVision) according to the manufacturer's protocol.

### 2.6. Statistical Analysis

Comparisons between groups were analyzed using the GraphPad Prism v.7.0 software (GraphPad Software, La Jolla, CA, USA). Continuous variables were expressed as the mean ± SD. Multiple groups were compared using one-way ANOVA followed by the Student-Newman-Keuls post hoc test. *P* < 0.05 was considered statistically significant.

## 3. Result

### 3.1. Characterization of Cultured ADSCs and EPCs

**Figure 1:**
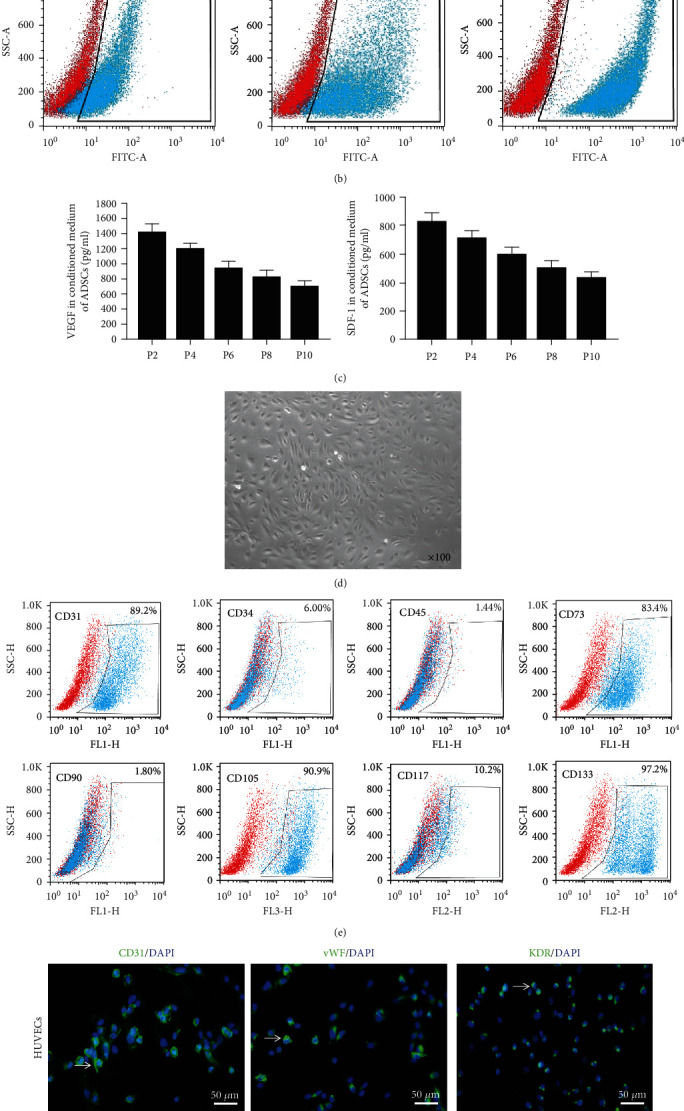
Characterization of cultured ADSCs and EPCs. (a) The adherent cultured rat ADSCs exhibited fibroblast-like morphology (×100) (b) Flow cytometry analysis demonstrated these ADSCs was strongly positive for CD73, CD90, and CD105. (c) The ELISA assay showed that ADSCs could secrete a large amount of VEGF and SDF-1 in vitro. (d) The cultured EPCs exhibited the typical cobblestone-shaped appearance (×100). (e) Flow cytometry analysis demonstrated these EPCs were strongly positive for CD31, CD73, CD105, and CD133. (f) Immunofluorescence staining confirmed that EPCs were positive for EC markers CD31, vWF, and KDR. Scale bars: 50 *μ*m. VEGF: vascular endothelial growth factor; SDF-1: stromal cell-derived factor-1; vWF: von Willebrand Factor; KDR: kinase insert domain receptor.

The adherent cultured rat ADSCs exhibited fibroblast-like morphology under an inverted phase contrast microscope. And the flow cytometry analysis demonstrated that these ADSCs were strongly positive for the MSC markers CD73, CD90, and CD105, as our previous study shown^20^ (Figures 1(a) and 1(b)). The ELISA assay showed that ADSCs could secret a large amount of VEGF and SDF-1, which have been proved to be important to the proliferation and differentiation of EPCs (Figure 1(c)).

The rat EPC colonies appeared 2 weeks after plating. These cells exhibited the typical cobblestone-shaped appearance (Figure 1(d)). The flow cytometry analysis demonstrated that these EPCs were positive for CD31, CD73, CD105, and CD133 (Figure 1(e)). Immunofluorescence staining confirmed that the cultured EPCs could express the HUVEC surface marker CD31, vWF, and KDR (Figure 1(f)).

### 3.2. ADSCs Enhance the Proliferation and Angiogenisis of EPCs In Vitro

**Figure 2:**
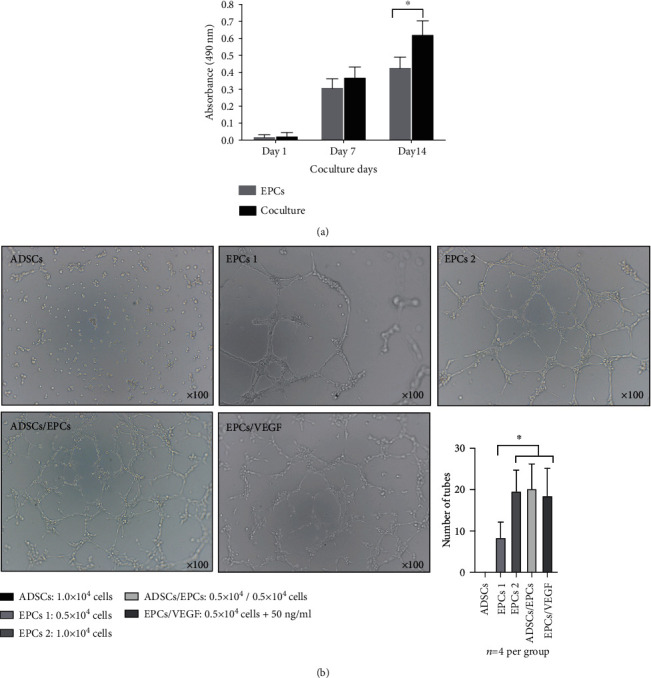
ADSCs enhanced the proliferation and angiogenesis of EPCs in vitro. (a) MTT assay showed that the number of EPCs cocultured with ADSCs was significantly more than EPCs cultured alone. ^∗^*p* < 0.05. *n* = 3 per group. (b) More tubes were formed on Matrigel by 0.5 × 10^4^/0.5 × 10^4^ ADSCs/EPCs, 1.0 × 10^4^ EPCs or 0.5 × 10^4^ EPCs+50 ng/ml VEGF compared to 0.5 × 10^4^ EPCs. ^∗^*p* < 0.05. *n* = 4 per group. MTT: 3-(4,5-dimethyl-2-thiazolyl)-2,5-diphenyl-2-H-tetrazolium bromide; VEGF: vascular endothelial growth factor.

Cultured in the transwell for 14 days, the number of EPCs co-cultured with ADSCs was significantly higher than that of EPCs cultured alone via the MTT assay (*p* < 0.05) (Figure 2(a)). More tubes were formed on Matrigel by 5 × 10^3^/5 × 10^3^ ADSCs/EPCs, 1 × 10^4^ EPCs, and 5 × 10^3^ EPCs plus 50 ng/ml VEGF, than 5 × 10^3^ EPCs (*p* < 0.05), while there was no significant difference among those three groups (*p* > 0.05) (Figure 2(b)).

### 3.3. Blood Glucose Concentration and Body Weight Do Not Change in the DED Rats after Cell Transplantation

After STZ intraperitoneal injection, blood glucose concentration was significantly increased and the body weight was significantly declined in the diabetic rats compared to the age-matched normal control rats (*p* < 0.05). 28 days after cell transplantation, the blood glucose level and body weight were not significantly change in the DED rats. Moreover, there were no DED rats died in this study (Table 1).

### 3.4. Combined Transplantation of EPCs and ADSCs Better Restores Erectile Function in the DED Rats

**Figure 3:**
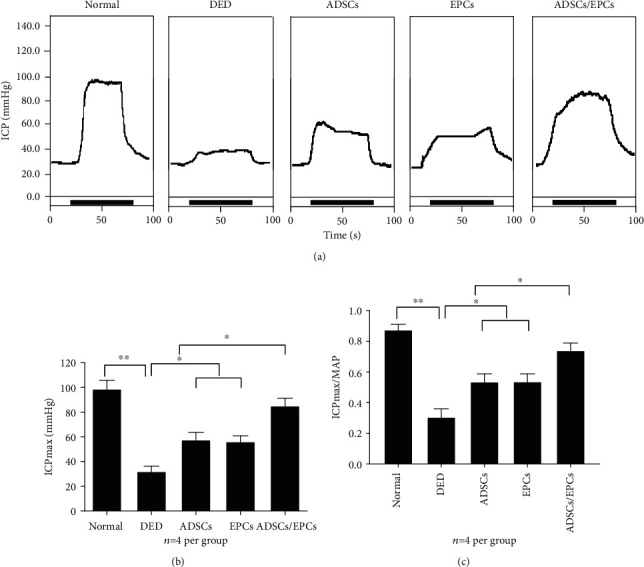
Combined transplantation of ADSCs and EPCs improved erectile function in the DED rats. (a) Representative ICP tracing response to the electronic stimulation of the cavernous nerve (5 V, 20 Hz, and 60 s duration) in DED rats after injection of PBS, ADSCs, EPCs, or ADSCs/EPCs (*n* = 4 rats per group). (b) The effects of ADSCs, EPCs, and ADSCs/EPCs on the increase of ICP in the DED rats. (c) The ratio of total ICP to MAP was calculated for all five groups. ^∗^*p* < 0.05, ^∗∗^*p* < 0.01. ICP: intracavernous pressure; MAP: mean arterial pressure; PBS: phosphate-buffered saline.

Only one rat in the EPC group died on day 22 after cell transplantation, and it was excluded in this study. Eight weeks after STZ injection, the ICP and ICP/MAP ratios were significantly decreased in the DED rats compared to the normal control rats (*p* < 0.01), indicating the impairment of erectile function in the DED rats. The ADSC group and the EPC group both showed greatly increased ICP and ICP/MAP ratios compared to the DED group but were still significantly lower than the normal control group (*p* < 0.05), demonstrating that ADSCs and EPCs both could partially restore the erectile function of DED rats. Meanwhile, ADSC/EPC cotransplantation displayed a greatly protective effect of erectile function. The total ICP and the ICP/MAP ratios of the ADSC/EPC group were 95 ± 9 mmHg and 69 ± 15%, respectively, which were higher than the values in the EPC group and the ADSC group and represented 86% and 90% of the values in the normal control group (Figures 3(a)-3(c)).

### 3.5. Combined Transplantation of ADSCs and EPCs Better Increases Cavernous Endothelial Function in the DED Rats

**Figure 4:**
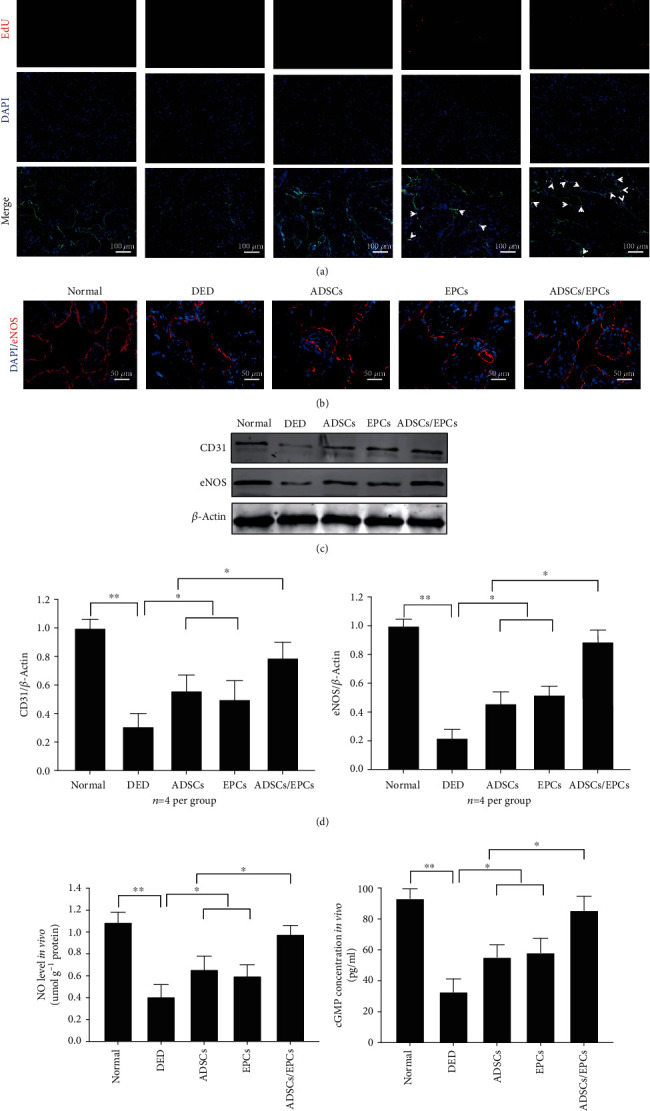
Combined transplantation of ADSCs and EPCs improved the endothelial function of DED rats. (a) Immunofluorescence staining of CD31 (green) and EdU (red) in the cavernous tissue in each group. The white arrow shows the transplanted EPCs. Scale bar: 100 *μ*m. (b) Immunofluorescence staining of eNOS (red) in the cavernous tissue in each group. Scale bar: 50 *μ*m (c, d) The representative images and quantity analysis of western blotting, including CD31 and eNOS. Data is expressed as relative expression compared to *β*-actin and to the normal group. (e) NO level in the serum of each group determined by ELISA assay. (f) cGMP concentration in the cavernous tissue in each group was determined by ELISA. ^∗∗^*p* < 0.01, ^∗^*p* < 0.05. EdU: 5-ethynyl-2′-deoxyuridine; eNOS: endothelial nitric oxide synthase; NO: nitric oxide; cGMP: cyclic guanosine monophosphate.

The endothelial marker CD31 expression level in the cavernous tissue 28 days after cell transplantation was determined by immunofluorescence staining and western blot (Figures 4(a), 4(c), and 4(d)). The result revealed that CD31 were significantly decreased in the DED rats compared to the normal control rats (*p* < 0.01), while combined transplantation of ADSCs and EPCs could almost completely restore the endothelial content in the cavernous tissue (*p* < 0.05). Meanwhile, ADSCs or EPCs could also partially recover the endothelial content, and there were no significant differences between these two groups (*p* > 0.05).  Figure 4(a) shows that more EdU-positive cells were found in the endothelial layer in the ADSC/EPC group than in the EPC group, though the number of transplanted EPCs in the ADSC/EPC group was just half of the EPC group, which implied that ADSCs could enhance the proliferation and differentiation of EPCs in vivo. Additionally, western blot confirmed that there were more CD31 expressed in the ADSC/EPC group than that in the EPC or ADSC group.

eNOS-NO-cCMP signaling was extremely important to endothelial function. Western blot showed a large decreased in eNOS expression in cavernous tissue of DED rats (Figures 4(b)-4(d)). In addition, NO concentration in serum and cGMP concentration in cavernous tissue of DED rats were also significantly lower than those in the normal rats. Expression level of eNOS, NO concentration, and cGMP concentration significantly increased 28 days after cell transplantation in the ADSC/EPC group than in ADSC, EPC, and DED group (*p* < 0.05), indicating that combined transplantation of ADSCs and EPCs could effectively restore the impaired eNOS-NO-cCMP signaling in DED rats (Figures 4(e) and 4(f)).

### 3.6. Transplanted ADSCs Increase VEGF and SDF-1 Expression Levels in Cavernous Tissue

**Figure 5:**
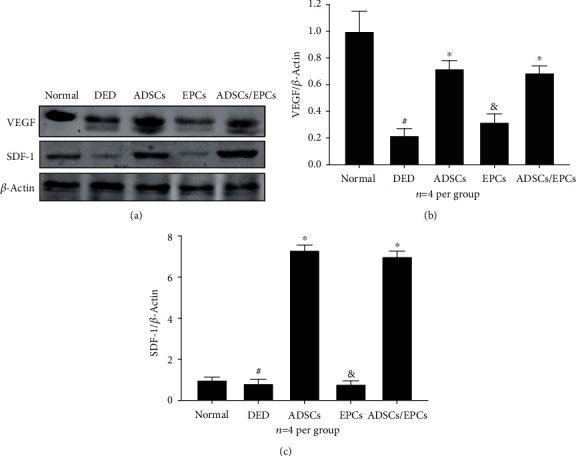
(a, b) The representative images of western blot of VEGF and SDF-1 in the cavernous tissue. (b, c) Quantitative analysis of western blotting, including VEGF and SDF-1. Data is expressed as relative expression compared to *β*-actin and to the normal group. #: compared with normal control, *p* < 0.05; ^∗^compared with the DED group, *p* < 0.05; &:compared with the DED group, *p* > 0.05. VEGF: vascular endothelial growth factor; SDF-1: stromal cell-derived factor-1.

The expression level of VEGF in cavernous tissue was significantly lower in the DED group than in the normal control group, when assessed with western blot, as our previous study has shown [8]. After intracavernous injection with ADSCs or ADSCs/EPCs, the expression level of VEGF was significantly increased within the cavernous tissue compared to the DED rats, while there were no differences between the EPC group and the DED group, suggesting that it was the paracrine effect of ADSCs but not EPCs which significantly increased the VEGF content in the cavernousum. Moreover, western blot demonstrated that the SDF-1 expression level in cavernous tissue in the ADSC group and the ADSC/EPC group was significantly higher than that other groups (*p* < 0.05) (Figures 5(a) and 5(b)).

### 3.7. Transplantation of ADSCs Alone or with EPCs Increases nNOS Expression in the Penile Dorsal Nerves in DED Rats

**Figure 6:**
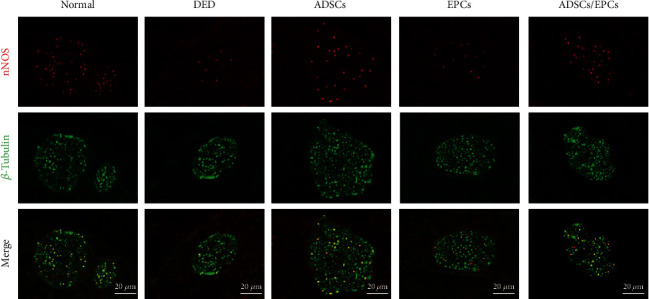
Transplantation of ADSCs or ADSCs/EPCs increased the nNOS-positive fibers in the penile dorsal nerve. Representative images of fluorescence costaining of nNOS (red) and *β*-tubulin (green) in the penile dorsal nerve. Scale bar: 20 *μ*m. nNOS: neuronal nitric oxide synthase.

Immunofluorescence staining demonstrated that the nNOS expression in the penile dorsal nerves in DED rats was significantly lower than that in the normal control rats (*p* < 0.05). After cell transplantation, rats in the ADSC group and the ADSC/EPC group both displayed higher nNOS expression in the penile dorsal nerves compared to the DED group (*p* < 0.05), while there was no significant difference of nNOS expression between the EPC group and the DED group (*p* > 0.05) (Figure 6).

### 3.8. Combined Transplantation of ADSCs and EPCs Better Increases Smooth Muscle Content in the Covernosa in DED Rats

**Figure 7:**
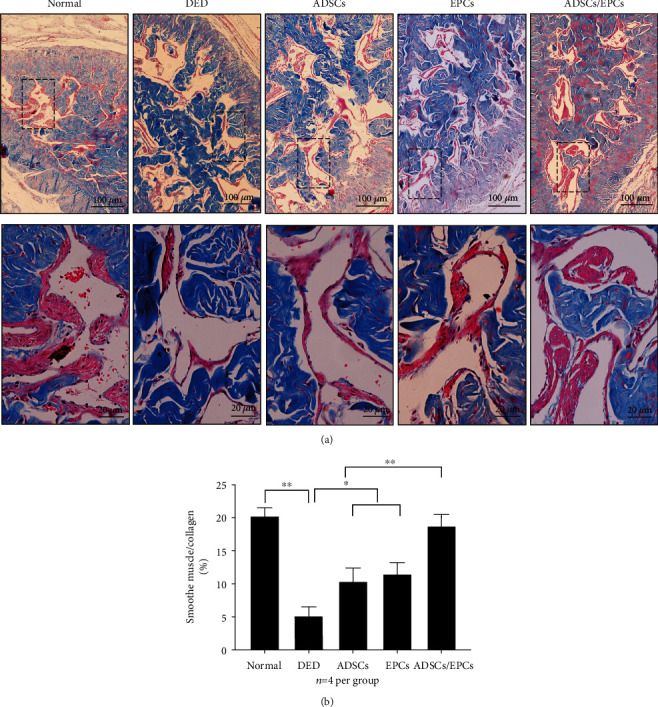
Combined transplantation of ADSCs and EPCs better increased smooth muscle content in the cavernosa in DED rats. (a) The representative images of Masson's trichrome staining of the cavernous tissue. Scale bar: 100 *μ*m and 20 *μ*m. (b) The smooth muscle-to-collagen ratio in the cavernous tissue was calculated in each experimental group. *n* = 5 per group; ^∗∗^*p* < 0.01, ^∗^*p* < 0.05.

Masson's trichrome staining showed that the SMC/collagen ratio was significantly decreased in the DED rats compared to the normal control rats (*p* < 0.01). Intracavernous injection of EPCs or ADSCs could improve the SMC/collagen ratio (*p* < 0.05), but it was still lower than that of the normal rats (*p* < 0.05). Interestingly, combined transplantation of ADSCs and EPCs could almost completely restore the SMC/collagen ratio (Figures 7(a) and 7(b)).

## 4. Discussion

Diabetic erectile dysfunction is closely associated with endothelial dysfunction, smooth muscle atrophy, and nerve degeneration [22]. The response rate to PDE5 inhibitor, such as sildenafil, is low in DED patients [3]. Studies have demonstrated that the number of EPCs in the peripheral blood of diabetic ED patients was significantly decreased compared to the healthy men, and the function of EPCs was also impaired, which indicated that EPCs might be involved in the pathogenesis of DED [23, 24]. Intracavernous injection of EPCs or ADSCs alone could partially restore the erectile function in the DED rat model [7, 16]. In this study, we found that cotransplantation of EPCs and ADSCs had a synergistic effect on repairing the endothelial function, thus significantly improved the erectile function in a STZ-induced diabetic ED rat model. Besides, similar to other researches [17, 18], cotransplantation of EPCs and ADSCs was safe that no rat in the ADSC/EPC group died in our study.

Studies have demonstrated that EPCs could be recruited to the impaired vessel and differentiate into vascular endothelial cells, thus repairing the vascular wall, which made it a promising strategy for the vascular regeneration [25]. Chronic administration of melatonin could prevent ED in the diabetic rats possibly via the mobilization of EPCs from the bone marrow [26]. In the present study, intracavernous injection of EPCs alone could partially restore the erectile function of DED rats by improving endothelial function. Our previous study found that the VEGF signaling system was impaired in the cavernous tissue of DED rats, which led to the endothelial dysfunction and erectile dysfunction [8, 27]. Researches have revealed that the proliferation and differentiation of EPCs required a number of proangiogenic factors, especially VEGF [28, 29]. The defected VEGF signaling pathway in the DED rats may explain the partial restoration effect of intracavernous injection of EPCs alone. Gao et al. showed that intracavernous injection of VEGF165-transfected EPCs could significantly restore erectile function of DED rats [16].

ADSCs, which could be obtained by minimally invasive method and have great potential of multidifferentiation and paracrine effect, are wildly used in the tissue regeneration region [30, 31]. Garcia et al. have first demonstrated that ADSCs could partially restore the erectile function of DED rats via the paracrine effect [7]. Furthermore, our previous study found that intracavernous injection of ADSCs genetically modified with VEGF could almost restore the erectile function of the DED rats, possibly via repairing the defected VEGF signaling system [8]. However, gene modification may have the risk of changing the host chromosome genome, which hinders its clinical application. Strassburg et al. showed that ADSCs could enhance the angiogenic potential of EPCs in vitro by secreting VEGF [32]. Our present study also found that ADSCs could significantly improve the proliferation of EPCs in vitro by transwell assay.

Cotransplantation of MSCs and EPCs have the synergistic effect in the treatment of cardiovascular disease [33] and cerebrovascular disease [34] and in the bone regeneration [35]. Fang et al. reported that combined periprostatic transplantation of MSCs and EPCs could significantly improve the erectile function in the cavernous never injury-induced ED rat model [36]. The mechanism might be MSCs could restore cavernous never by secreting neurotrophin factors containing NGF and VEGF, while EPCs could enhance the paracrine effect of MSCs. In our present study, the ICP and ICP/MAP ratio results revealed that intracavernous injection of 5 × 10^5^/5 × 10^5^ ADSCs/EPCs could nearly almost restore the erectile function of DED rats, while transplantation of 1 × 10^6^ ADSCs or 1 × 10^6^ EPCs alone only partially restored the erectile function, which indicated the synergistic effect of ADSCs and EPCs in the treatment of DED. Moreover, nNOS expression in the ADSC group and the EPC/ADSC group was significantly higher than the PBS group and the EPC group, indicating the neuroprotective effect of ADSCs. But in Fang's study, cells were transplanted periprostatically, indicating that the therapeutic effect of ADSCs and/or EPCs was mostly via the paracrine activity. While in our research, stem cells were transplanted into the corpus cavernosum, making it possible to participate directly in the repair of cavernous tissue, especially the impaired endothelium.

We only labeled the EPCs with EdU in this study for the reason that others and our previous study all demonstrated that ADSCs could not be found in the cavernous tissue after 28-day transplantation. EdU-positive cells could be found lining in the endothelial layer in the cavernosum 28 days after cell transplantation. Interestingly, the total number of transplanted EPCs in the EPC/ADSC group was just half of the EPC group, but significantly more EdU-positive cells were found in the EPC/ADSC group than the EPC group, which could be partially attributed to the paracrine of VEGF by the cotransplanted ADSCs. ADSCs can paracrine not only proangiogenic factors but also stromal cell-derived factor 1 (SDF-1), which is a well-characterized cytokine that regulates the recruitment of EPCs to the sites of neo-angiogenic niches in the injured tissues [37–39]. Western blot results displayed high SDF-1 expression which was found in cavernous tissue in the ADSC group and the EPC/ADSC group, which might explain why more EdU-positive cells were found in the EPC/ADSC group compared to the EPC group. SDF-1 improves the mobilization, migration, homing, and vasculogenesis of EPCs under hyperglycemia via activating the SDF-1/CXCR4 axis [10]. Moreover, research even found that VEGF and SDF-1 had a synergic effect on the angiogenic property of EPCs [40]. We speculate that SDF-1 might also recruit endogenous EPCs to the cavernousm to repair damaged endothelial function, but it needs more investigation.

There were several limitations in the current research. First, we could not identify whether the CD31 and EdU double-positive cells in the endothelium were EPCs or mature ECs, for both of them express CD31, and there is no single marker which could distinguish between them at present. Second, the study demonstrated that stem cell therapy may have a long-term effect in the treatment of pelvic neurovacular injury-induced ED [41]. Considering that hyperglycemia may affect the function of ADSCs and/or EPCs over time, the long-term efficacy of EPCs and ADSCs cotransplantation needs a long-term follow-up. Third, the optimal amount and proportion of transplanted ADSCs and EPCs need further study.

## 5. Conclusion

In the present study, we demonstrate that intracavernous cotransplantation of EPCs and ADSCs has a synergic effect in repairing the endothelial function, which significantly improves the erectile function in a type 2 diabetic ED rat model. The underling mechanism might be the paracrine of VEGF and SDF-1 by ADSCs improves the recruitment and proliferation of EPCs in the cavernosum. Moreover, cotransplantation can also increase the smooth muscle and nNOS expression in the cavernosum in the DED rat model. Therefore, cotransplantation of EPCs and ADSCs might provide novel therapeutic opportunity for DED patients.

## Figures and Tables

**Table 1:** The body weight and fasting glucose before and after cell transplantation in each group. Data are expressed as *mean* ± *standard* deviation from *N* = 14 per group. ^∗^*p* < 0.05 vs the control group. ▵*p* < 0.05 vs the DEM group.

Variable	Normal (*n* = 9)	DED (*n* = 9)	ADSCs (*n* = 9)	EPCs (*n* = 9)	ADSCs/EPCs (*n* = 9)
Initial					
Body weight(g)	288.56 ± 22.13	290.13 ± 20.26	296.76 ± 21.22	302.54 ± 20.19	288.89 ± 19.07
Fasting glucose (mmol/L)	5.78 ± 0.49	5.82 ± 0.52	5.76 ± 0.50	5.88 ± 0.61	5.91 ± 0.43
Final (after 12 weeks)					
Body weight(g)	589.35 ± 41.32	225.12 ± 18.72^∗^	230.66 ± 19.13^∗▵^	239.82 ± 20.45^∗▵^	229.46 ± 22.78^∗▵^
Fasting glucose (mmol/L)	5.83 ± 0.51	27.71 ± 2.45^∗^	28.32 ± 1.69^∗▵^	28.11 ± 2.02^∗▵^	27.30 ± 1.95^∗▵^

## Data Availability

The data are available by contacting the corresponding authors.
